# Two more new species of *Aphidura* (Hemiptera, Aphididae), and a note on variation in *Aphidura bozhkoae* Narzikulov

**DOI:** 10.3897/zookeys.425.7797

**Published:** 2014-07-15

**Authors:** Juan M. Nieto Nafría, Roger L. Blackman, Jon H. Martin

**Affiliations:** 1 Departamento de Biodiversidad y Gestión Ambiental; Universidad de León; 24197 Leon (Spain); 2 Department of Life Sciences, Natural History Museum; Cromwell Road; London (United Kingdom)

**Keywords:** *Aphidura*, new taxa, descriptions, intraspecific variation, key of species

## Abstract

Two new species of *Aphidura* Hille Ris Lambers, 1956 (Hemiptera, Aphididae, Macrosiphini) are described; *Aphidura libanensis* **sp. n.** from *Prunus prostrata* in Lebanon, and *Aphidura corsicensis* **sp. n.** from *Cerastium soleirolii* in Corsica (France). Studies of *Aphidura bozhkoae* specimens from different localities have revealed that this species varies in its pattern of dorsal sclerotisation and other morphological characters, within and between populations. An updated key for identifying the world’s species of *Aphidura* is presented.

## Introduction

*Aphidura* Hille Ris Lambers, 1956 (Hemiptera, Aphididae, Macrosiphini) currently includes 22–25 subjective valid species, exhibiting a Mediterranean-Pontian-Turanian distribution with extensions to neighbouring areas and exceptionally (one species) to the Russian Far East. Many of the species are associated with Caryophyllaceae, and some feed on *Prunus* and related genera, indicating that there may be host alternation between Amygdaloideae (Rosaceae) and Caryophyllaceae, but their biology and life cycles are mostly unknown (Blackman and Eastop 2014). Twelve of the species were described in 2013 (Kadyrbekov 2013; Nieto Nafría et al. 2013), adding greatly to the knowledge of the genus. Two more species were present among specimens conserved in the collection of the Natural History Museum in London (BMNH), and they are described in this paper.

*Aphidura bozhkoae* Narzikulov, 1958 was described from *Prunus* (= *Cerasus*) *verrucosa* in Tajikistan, and has subsequently been found on various species of *Prunus* in the neighbouring countries Kyrgyzstan and Uzbekistan, and also in Kazakhstan, Iran and Georgia (Nieto Nafría et al. 2013). The variation in several morphological features exhibited by this species, both within and between populations, is remarkable. In particular, Iranian specimens (illustrated by Nieto Nafría et al. 2013: 10, fig 3A) differ from the Tajik type series (illustrated by Narzikulov 1958: 16, fig. 1) to such an extent that D. Hille Ris Lambers gave them the specific epithet “nitens”, which was however never published. The variable characters include those that have been used to discriminate between other species of *Aphidura*, some of which are described from only single collections, so for future work on this genus it is important that the variation in *Aphidura bozhkoae* should be reviewed and discussed.

## Material and methods

Specimens of the following five samples have been studied:

(1) 9 apterous viviparous females and 1 alate viviparous female: TAJIKISTAN: *Cerasus verrucosa*, Iskandirkul, 14-VII-1959 (3 apterae, 1 alate); same host-plant, Ziddy [Siddi in slide label by Hille Ris Lambers], 28-VI-1954 (6 apterae), N. Narzikulov *leg.*; *Aphidura bozhkoae*, Narzikulov *det.*; BMNH collection. Both Tajik localities are type-localities of this species (Narzikulov 1958: page 20).

(2) 54 apterous viviparous females and 13 alate viviparous females: TAJIKISTAN: *Cerasus verrucosa*, Varzov (1 km above Kondara), 24-V-1981; *Cerasus erythrocarpa*, Ziddy, 9-VI-1981, G. Kh. Shaposhnikov *leg.* (samples 7524 and 7551); *Aphidura bozhkoae*, G. Kh. Shaposhnikov *det.*; *Zoologicheskiy Institut* collection. Ziddy is a type-locality of this species and Varzov is near Kondara, which is also a type-locality. These specimens were dry (alcohol had completely evaporated); they were hydrated and later mounted on microscopic slides.

(3) 115 apterous viviparous females and 63 alatae viviparous females: IRAN: several host plants, localities and dates (data in Nieto Nafría et al. 2013: table I); G. Remaudière *leg.* (samples i196, i2961, i3028, i4092 and i4347); *Aphidura* “nitens”, manuscript name, D. Hille Ris Lambers or G. Remaudière *det.*; *Aphidura bozhkoae*, J. M. Nieto Nafría *det.*. *Muséum national d’Histoire naturelle* and BMNH collections.

(4) 5 apterous females: LEBANON, *Prunus prostrata*, Jabal el Barouk, 22-V-1973, D. Hille Ris Lambers *leg.* (sample 754); *Aphidura* “nitens” manuscript name, D. Hille Ris Lambers *det.*; BMNH collection.

(5) 3 apterous viviparous females: CORSICA (France), *Cerastium soleirolii*, North slopes of Mount Cinto, 08-VII-1980, J. H. Martin *leg.* (sample number 3061); *Aphidura* “cerastii”, manuscript name, D. Hille-Ris-Lambers *det.*; BMNH collection.

Morphological measurements were made according to Nieto Nafría and Mier Durante (1998). Measurements in text and table are lengths except when indicated otherwise as width or diameter. Tables have a distribution of data like tables in Nieto Nafría et al. (2013) in order to facilitate the comparisons.

A Leica DC digital 96 camera with IM 1000 version 1.10 software was used for the photomicrographs, which have been taken and mounted by L. M. Fernández Blanco.

## Results and discussions

### Descriptions of new species

#### Aphidura libanensis sp. n.

Taxon classificationAnimaliaHemipteraAphididae

http://zoobank.org/ECE4A97D-D03F-45CA-BEB5-57C64853ED30

##### Diagnosis.

Siphunculi tapering to apex or slightly swollen in a distal portion. Dorsum of metathorax to abdominal segment 6 with a discal plate (apterae). Mesosternal mammariform processes pale, flat and with spinules (apterae). Abdominal marginal tubercles usually present. Tarsal formula 3.3.3. (sometimes one tarsus with 4 setae)

##### Apterous viviparous female

**Figure 1.**
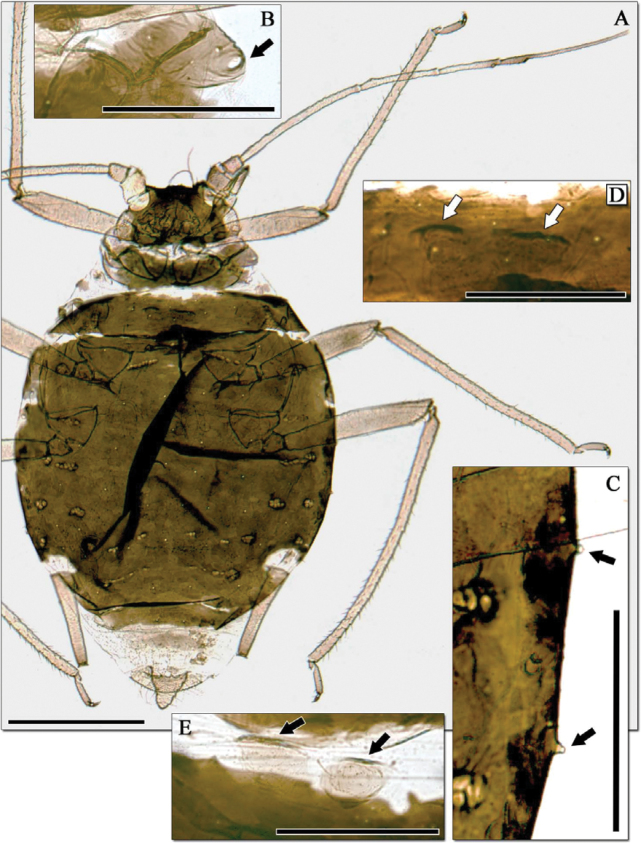
*Aphidura libanensis* sp. n., apterous viviparous females. **A** Habitus **B** Part of prothorax with a marginal tubercle (signalled with an arrow) **C** Marginal zone of abdominal segments 3 and 4, with marginal tubercles (signalled with arrows) **D** Mesosternum with mammariform processes (signalled with arrows) **E** Mesosternum with mammariform processes (signalled with arrows) of another specimen.

from 5 specimens (Figs 1A–E). Unknown colour in life, possibly shiny black with antennae, legs and siphunculi pale brown. In mounted specimens, antennae, mesosternal mammariform processes, legs, siphunculi, abdominal segment 8 sclerotized band, cauda and genital and anal plates are yellowish brown, with very proximal and distal portions of siphunculi, antennal segment VI and very apical portion of antennal segment V somewhat darker than aforementioned structures, all in contrast with head and dorsum of thorax and most part of abdomen, which are very dark brown. Frons undulated. Head with rugosity lines and spinules (both more abundant on dorsum). Antennal cuticle imbricated. Setae on body dorsum, antennae, and most of those on legs thick, with apices blunt or slightly capitate. Mesosternal mammariform processes pale, flat and with spinules. A discal plate present (from metathorax to abdominal segment 6); prothorax, mesothorax and abdominal segment 7 with wide transverse bands, which are darker than the discal plate, abdominal segment 8 with a band paler than the others. Marginal tubercles usually present on both sides of prothorax and several abdominal segments; they are small, but the abdominal ones are relatively tall. Siphunculi tapering to apex or slightly swollen in the distal quarter, spinulose imbrication, distinct preapical incision and flange. Cauda broadly triangular. Metric and meristic features in Table 1.

**Table 1.** Metric and meristic features of apterous viviparous females of *Aphidura libanensis* sp. n. and *Aphidura corsicensis* sp. n.

	*Aphidura libanensis*	*Aphidura corsicensis*
	n=5	n=3
Body [mm]	1.805–1.938	2.025–2.088
Antenna [mm]	1.605–1.698	1.590–1.655
Antenna / Body [times]	0.87–0.93	0.79–081
Ant. segm. III [mm]	0.43–0.51	0.48–0.52
Ant. segm. IV [mm]	0.27–0.31	0.27–0.29
Ant. segm. V [mm]	0.20–0.24	0.22–0.24
Ant. segm. VI base [mm]	0.09–0.13	0.11–0.12
Ant. segm. VI processus terminalis	0.39–0.46	0.31–0.34
Ant. segm. VI processus terminalis / Ant. segm. III [times]	0.78–1.03	0.60–0.67
Ant. segm. VI processus terminalis / base [times]	3.56–4.89	2.63–3.00
Ultimate rostral segm. [mm]	0.12–0.13	0.15–0.16
Ultimate rostral segm. / its basal width [times]	2.00–2.27	2.58–2.73
Ultimate rostral segm. / Ant. segm. VI base [times]	0.96–1.39	1.25–1.36
Hind femur [mm]	0.52–0.56	0.61–0.63
Hind tibia [mm]	0.96–1.03	1.06–1.09
Hind tibia / Body [times]	0.52–0.56	0.52–0.53
Hind tarsus, 2nd segm. [mm]	0.11–0.12	0.10–0.11
Hind tarsus, 2nd segm. / Ultimate rostral segm. [times]	0.84–1.00	0.65–0.75
Abdominal (segm. 2–6) marginal tubercles [number]	0–3	0
Siphunculus [mm]	0.30–0.35	0.42–0.45
Siphunculus / Body [times]	0.17–0.18	0.20–0.22
Siphunculus / Ant. segm. III [times]	0.65–0.74	0.82–0.89
Siphunculus / its basal width [times]	3.50–4.27	4.83–5.60
Siphuncular width, maximal / basal [times]	0.56–0.70	0.61–0.82
Siphuncular width, maximal / minimal [times]	1.05–1.11	1.04–1.10
Siphuncular minimal width / Hind tibiae, diameter at middle [times]	1.13–1.80	1.00–1.18
Cauda [mm]	0.13–0.16	0.23–0.24
Cauda / siphunculus [times]	0.40–0.49	0.51–0.57
Cauda / its basal width [times]	0.87–0.97	1.41–1.50
Setae on...		
... Frons [μm]	25–35	32–37
... Frons / b. d. Ant. segm. III [times]	1.1–1.6	1.3–1.5
... Vertex [μm]	15–30	32–35
... Vertex / b. d. Ant. segm. III [times]	0.7–1.4	1.2–1.4
... Antennal segment III [μm]	12–15	10–14
... Antennal segment III / b. d. Ant. segm. III [times]	0.6–0.7	0.4–0.6
... Ultimate rostral segm, [number]	6–9	17–19
... Hind femur, dorsal [μm]	12–18	17–25
... Hind femur, ventral [μm]	22–28	25–38
... Hind tibia, dorsal, at middle [μm]	22–33	27–33
... Hind tibia, dorsal / Tibial diameter (at middle) [times]	0.5–0.8	0.5–0.7
... Hind tarsus, first segm. [number]	3 (4)	3
... Abdominal segm. 2–4 [μm]	10–18	25–30
... Abdominal segm. 2–4 / b. d. Ant. segm. III [times]	0.5–0.8	1.0–1.1
... Abdominal segm. 8 [μm]	27–33	32–38
... Abdominal segm. 8 / b. d. Ant. segm. III [times]	1.2–1.5	1.3–1.4
... Abdominal segm. 8 [number]	4	3–4
... Genital plate, discal [number]	2	5–7
... Genital plate, marginal [number]	10–15	13–20
... Cauda [number]	6–8	6–9

Note: Used abbreviations: Ant., Antennal; b. d., basal diameter; n. number of measured specimens; segm., segment or segments.

##### Types.

Holotype: apterous viviparous female, collected on *Prunus prostrata* Labill. (Rosaceae), Jabal el Barouk (Lebanon), 2-V-1973, Hille Ris Lambers *leg.*; paratypes: four apterous females collected at the same time as the holotype.

##### Etymology.

The specific name of the new species is an adjective that means inhabitant of Lebanon, in feminine.

##### Discussion.

D. Hille Ris Lambers thought that these Lebanese specimens were conspecific with others found on *Prunus* in Iran, which were being studied by him and G. Remaudière, and that they all belonged to an undescribed species, which was named in draft “nitens” (Remaudière's epistolary archive). Certainly these Lebanese aphids do not belong to any previously described species, but they are also not conspecific with the Iranian ones, which we believe to be *Aphidura bozhkoae* (see next section).

*Aphidura libanensis* sp. n. resembles Iranian specimens of *Aphidura bozhkoae* and the East Asian species *Aphidura mordvilkoi* in the shape of the siphunculi and the extensive and solid discal plate, but there is an important difference in the number of first tarsal setae: four setae on each first tarsal segment in *Aphidura bozhkoae* and *Aphidura mordvilkoi*, and three in *Aphidura libanensis*. *Aphidura libanensis* and *Aphidura mordvilkoi* can also be distinguished from one another by the number of accessory setae on the ultimate rostral segment (6–9 in *Aphidura libanensis*, 2–4 in *Aphidura mordvilkoi*) and the relative length of the processus terminalis (3.6–4.9 times base of antennal segment VI in *Aphidura libanensis*, 2.2–2.7 times in *Aphidura mordvilkoi*). *Aphidura libanensis* and *Aphidura bozhkoae* are very similar in absolute and relative lengths of most body parts, the setae of *Aphidura libanensis* (Table 1) are all generally longer than those of *Aphidura bozhkoae* (Table 2).

**Table 2.** Metric and meristic features of apterous viviparous females of *Aphidura bozhkoae* from Tajikistan, collected by Narzikulov (column Tajik. [Na.]) and by Shaposhnikov (column Tajik. [Sh.], and from Iran, collected by Remaudière. In parenthesis exceptional data.

	Tajik. [Na.]	Tajik. [Sh.]	Iran [Re.]
	n = 7	n = 13	n = 31
Body [mm]	1.950–2.125	1.925–2.250	1.413–2.125
Antenna [mm]	1.408–1.748	1.475–1.785	1.290–1.765
Antenna / Body [times]	0.70–0.83	0.70–0.88	0.73–0.92(1.07)
Ant. segm. III [mm]	0.36–0.49	0.40–0.48	0.35–0.52
Ant. segm. IV [mm]	0.22–0.28	0.22–0.28	0.19–0.34
Ant. segm. V [mm]	0.19–0.22	0.18–0.24	0.18–0.25
Ant. segm. VI base [mm]	0.10–0.12	0.10–0.12	0.09–0.12
Ant. segm. VI processus terminalis	0.39–0.52	0.40–0.55	0.33–0.48
Ant. segm. VI processus terminalis / Ant. segm. III [times]	0.98–1.10	0.98–1.18	(0.72)0.85–1.15
Ant. segm. VI processus terminalis / base [times]	3.58–4.76	3.86–5.00	2.89–4.55
Ultimate rostral segm. [mm]	0.12–0.13	0.13–0.15	0.11–0.14
Ultimate rostral segm. / its basal width [times]	1.56–2.36	1.92–2.64	1.92–2.89
Hind tibia / Body [times]	0.44–0.52	0.42–0.55	0.47–0.62
Hind tarsus, 2nd segm. [mm]	0.12–0.14	0.12–0.14	0.10–0.13
Hind tarsus, 2nd segm. / Ultimate rostral segm. [times]	0.92–1.04	0.89–1.00	0.77–1.00
Abdominal marginal (segm. 2–6) tubercles [number]	0	0	0–6
Abdominal (segm. 8) spinal tubercles [number]	0	0	0(2–3)
Siphunculus [mm]	0.35–0.48	0.36–0.45	0.28–0.44
Siphunculus / Body [times]	0.17–0.22	0.18–0.22	0.17–0.25
Siphunculus / Antennal segm. III [times]	0.90–1.04	0.85–1.10	0.69–0.92
Siphunculus / its basal width [times]	3.68–5.67	3.60–5.25	3.42–6.15
Siphuncular width, maximal / basal [times]	0.60–0.73	0.50–0.75	0.53–0.85
Siphuncular width, maximal / minimal [times]	1.00–1.04	1.00–1.10	1.00–1.11
Siphuncular minimal width / Hind tibiae, diameter at middle [times]	1.14–1.50	1.10–1.60	1.06–1.64(2.00)
Cauda [mm]	0.17–0.18	0.17–0.22	0.12–0.18
Cauda / siphunculus [times]	0.37–0.51	0.39–0.51	0.35–0.48
Cauda / its basal width [times]	1.08–1.21	1.05–1.33	0.87–1.33
Setae on...			
... Frons [μm]	10–14	7–20	8–13
... Frons / b. d. Ant. segm. III [times]	0.3–0.5	0.3–1.0	0.3–0.6
... Vertex [μm]	10–15	10–22	8–10
... Vertex / b. d. Ant. segm. III [times]	0.5–0.6	0.4–1.0	0.3–0.6
... Antennal segment III [μm]	7–10	7–10	6–10
... Antennal segment III / b. d. Ant. segm. III [times]	0.3–0.4	0.3–0.4	0.3–0.5
... Ultimate rostral segm, [number]	9–13	7–18	6–11
... Hind femur, dorsal [μm]	10–13	9–18(28)	8–13
... Hind femur, ventral [μm]	12–20	12–20(35)	10–23
... Hind tibia, dorsal, at middle [μm]	15–24	17–30	10–23
... Hind tibia, dorsal / Tibial diameter (at middle) [times]	0.4–0.6	0.4–0.8	0.2–0.8
... Hind tarsus, first segm. [number]	4	4	4
... Abdominal segm. 2–4 [μm]	9–13	10–15	(5)8–10
... Abdominal segm. 2–4 / b. d. Ant. segm. III [times]	0.3–0.5	0.4–0.7	0.2–0.5
... Abdominal segm. 8 [μm]	10–15	12–20(25)	8–15
... Abdominal segm. 8 / b. d. Ant. segm. III [times]	0.4–0.6	0.6–0.9(1.1)	0.3–0.6
... Abdominal segm. 8 [number]	2–4	(2)4(5)	4
... Genital plate, discal [number]	2(4)	2(3)-	2
... Genital plate, marginal [number]	12–20	12–24	12–19
... Cauda [number]	8–12	7–10	6–10

Note: Used abbreviations: Ant., Antennal; b. d., basal diameter; n. number of measured specimens; segm., segment or segments.

#### Aphidura corsicensis sp. n.

Taxon classificationAnimaliaHemipteraAphididae

http://zoobank.org/EA353AA4-B640-4294-A9B0-8A7C58C546CB

##### Diagnosis.

Siphunculi slightly swollen. Dorsum of thorax and abdomen with setiferous sclerites (apterae). Mesosternal mammariform processes pale, flat and smooth (apterae). Abdominal marginal tubercles absent. Tarsal formula 3.3.3

###### Apterous viviparous female

**Figure 2.**
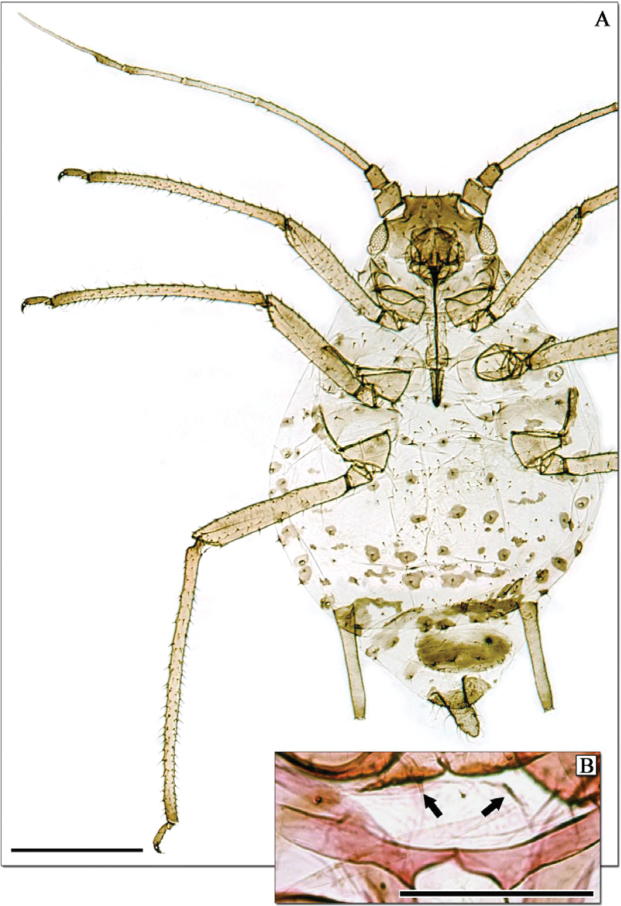
*Aphidura corsicensis* sp. n., apterous viviparous female. **A** Habitus **B** Mesosternum with mammariform processes (signalled with arrows).

from 3 specimens (Fig. 2). Shiny dark green in life. In mounted specimens, head, antennae, legs, siphunculi, cauda, genital and anal plates, and thoracic and abdominal sclerites yellowish brown, with apex of antennal segments III, IV and V, antennal segment VI, very apex of tibiae, tarsi and apex of siphunculi something darker than aforementioned structures. Frons undulated. Head mostly smooth, some rugosity lines are present on dorsum. Proximal section of antennal segment III and segments IV-VI imbricated. Setae on body dorsum, antennae and most of those on legs thick with apices blunt or slightly capitate. Mesosternal mammariform processes pale, flat and smooth. Setiferous sclerites present on dorsum of thorax and abdomen, mostly with one setae and similar in size to spiracular sclerites; marginal sclerites on abdominal segments 2-4 often coalescent; those on segment 6 forming postsiphuncular sclerites; and those on segment 7 and 8 partially coalescent; sclerites on abdominal segments 6-8 with spinules. Intersegmental sclerites small. Siphunculi slightly swollen (maximal width 1.04-1.10 times minimal width of the stem), with small scales and distinct preapical incision and flange. Cauda tongue shaped. Metric and meristic features in Table 1.

##### Types.

Holotype: apterous viviparous female, collected on *Cerastium soleirolii* Ser. ex Duby. (Caryophyllaceae), North slope of Mount Cinto, (Corsica, France), noted as more than 1100m, 08-VII-1980, J. H. Martin *leg.*. Paratypes: two apterous females from the same colony as the holotype.

##### Etymology.

The specific name of the new species is an adjective that means inhabitant of Corsica, in feminine.

##### Discussion.

This species was included as *Aphidura* sp. in the host lists and key to aphids on *Cerastium* by Blackman and Eastop (2006), but has not previously been formally described.

*Aphidura corsicensis* can be easily distinguished from other *Aphidura* species by the abundant dorsal setiferous sclerites, which are not present in any other species of the genus. The host plant is not normally found below 1900m (Arthur Chater, Kew Gardens, pers. comm.) but the plant hosting *Aphidura corsicensis* was estimated to have been collected at around 1100-1200m (Martin).

### The variability of *Aphidura bozhkoae* Narzikulov

Narzikulov (1958) described *Aphidura bozhkoae* as a shiny black aphid with black siphunculi feeding on *Cerasus verrucosa* (currently in *Prunus*) in Tajikistan. His illustration (Fig. 1 on p.16) depicts an aptera with extensive dorsal sclerotisation, but the metanotal sclerite is separate from the abdominal discal plate and has a mesial gap. In specimens from the type series in the BMNH collection this gap is quite wide and coincides with an indentation of the anterior part of the abdominal discal plate, to form a conspicuous irregularly-shaped window. All these specimens have siphunculi as dark as or darker than the discal plate. In contrast, specimens from Iran in the BMNH collection all have the metanotal sclerite forming a broad band fused with the abdominal discal plate. so that the sclerotisation extends as a solid shield from metonotum to abdominal tergite 6. Furthermore, these apterae have pale siphunculi. Another difference between Tajik and Iranian specimens in the BMNH collection concerns the mesosternal processes, which are well developed and well separated from each other in Tajik apterae, but much lower and more rounded in the specimens from Iran. These last features, all of them, are also shown by the specimen in Fig. 2A of Nieto Nafría et al. 2013).

In addition, the Tajik specimens all lack marginal or spinal tubercles, whereas in specimens from Iran small marginal tubercles may be present on some of abdominal tergites 2-6, and there may also be spinal tubercles on abdominal tergite 8 (Table 1).

**Figure 3.**
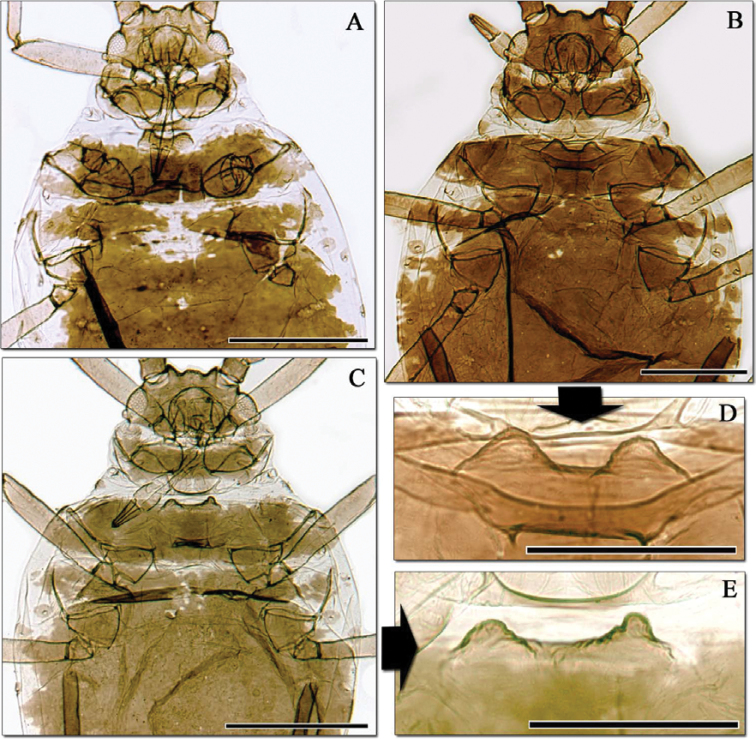
*Aphidura bozhkoae* apterous viviparous females from Ziddy (Tajikistan). **A–C** habitus, anterior part **D–E** Mesosternum with mammariform processes. **A** specimen collected in 1954 by Narzikulov **B–E** Two specimens collected in 1981 by Shaposhnikov. Note the different dorsal pattern and the different size and form of processes.

**Figure 4.**
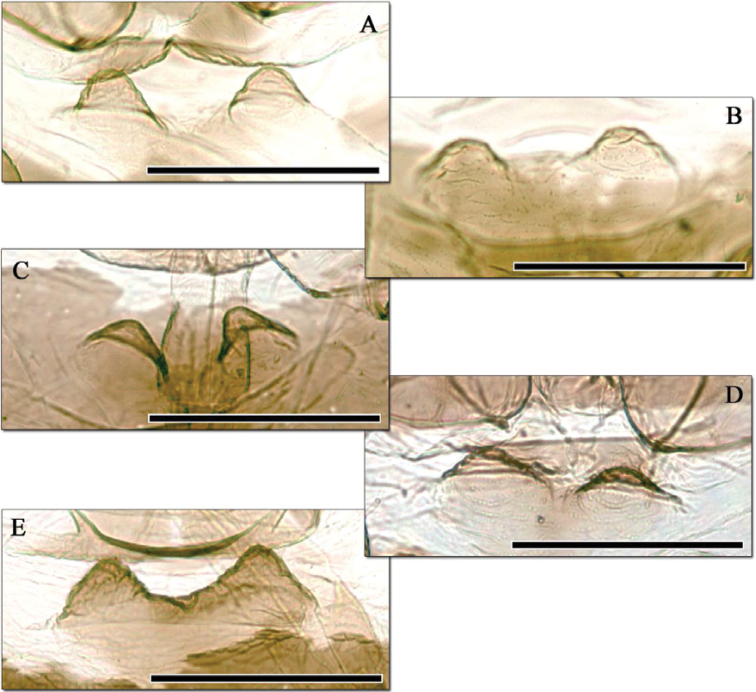
*Aphidura bozhkoae* mesosternal processes of five apterous viviparous females collected in Ziddy (Tajikistan) in 1981 by G. Shaposhnikov, same sample as the two specimens in Figure 3.

When morphometric data are compared, however, the ranges of measurements and ratios for all characters agree well between countries, any slight differences being within the expected range of intraspecific variation (Table 2). Furthermore, examination of more Tajik material from the *Zoologicheskiy Institut* collection (Figs 3, 4), and of more apterae from Iran in the collection of the *Muséum national d’Histoire naturelle*, showed that the BMNH specimens constituted the extremes of a range of variation in a single species, with Iranian apterae in particular varying in the dorsal pattern of sclerotisation, pigmentation of siphunculi and shape of mesosternal processes. Further confirmation came from photomicrographs of *Aphidura bozhkoae* from *Prunus incana* in Georgia kindly provided by S. Bardjadze, which showed a similar range of variation in these characters within a single sample.

Intraspecific variation in the extent of dorsal sclerotisation is evident in other species of *Aphidura*, especially in those that are widely collected such as *Aphidura picta*. However this usually involves varying degrees of fragmentation or reduction of the entire discal plate rather than any specific part of it; in this respect the variation shown by *Aphidura bozhkoae* is unusual. Siphuncular pigmentation and the extent of development of the mesosternal processes are characters that have been used to discriminate between *Aphidura* species, but experience with *Aphidura bozhkoae* shows that they need to be applied carefully, especially when describing new species from small samples.

### Key to apterous viviparous females of *Aphidura* species of the world (updated from the key of Nieto Nafría et al. 2013).

In brackets are: (1) when necessary morphological characters are included that do not have correspondence in the other proposition of the disjunctive, but which are useful to confirm identification; (2) illustration reference, from literature or in this paper; (3) host plants; and (4) distribution, with countries listed in geographical order from West to East.

**Table:** 

1	Siphunculi markedly swollen (maximal swollen width at least 1.2 times minimal stem width)	2
–	Siphunculi of different form (cylindrical, subcylindrical, tapering or slightly swollen, see Nieto Nafría et al. (2013): “generic characters” section)	9
2	Most of dorsal setae placed on conical tubercles. [Dorsum without segmental pigmented sclerotisation; Nieto Nafría et al. (2013): fig. 6. On *Acanthophyllum* sp. Iran]	*Acanthophyllum acanthophylli*
–	Dorsal setae not placed on tubercles	3
3	Mesosternal processes and cauda pale	4
–	Mesosternal processes and cauda more or less pigmented, light brown to brown	6
4	Siphunculi dark brown, 2.3–2.7 times cauda which has 7–11 setae. Abdominal dorsum with spino-pleural patch, postsiphuncular sclerites pigmented and marginal sclerites. [Ultimate rostral segment 1.0–1.2 times second segment of hind tarsi; cauda 1.1–1.2 times its basal width; Kadyrbekov (2013): fig. 8. On *Silene suffrutescens* and *Silene* sp. Kazakhstan]	*Aphidura nomadica*
–	Siphunculi pale, sometimes with smoky apex, 1.6–2.2 times cauda, which has 6–7 setae. If a spino-pleural patch present then ultimate rostral segment is 1.2–1.5 times second segment of hind tarsi	5
5	Antennal segment VI processus terminalis at least 1.4 times antennal segment III and approximately 4 times antennal segment VI base. Longest dorsal setae on abdominal segment 2–4 approximately 3 μm. Cauda tongue-shaped. Dorsum pale with dark intersegmental sclerites. [Nieto Nafría et al. (2013): fig. 5C. On *Gypsophila* sp. Grece]	*Aphidura graeca*
–	Antennal segment VI processus terminalis at most 1.1 times antennal segment III and at most 3.1 times antennal segment VI base. Longest dorsal setae on abdominal segment 2–4 are 7–13 μm. Cauda triangular, sometimes slight constricted. Dorsum with variable sclerotisation and pigmentation, sometimes mostly pale. [Nieto Nafría et al. (2013): fig. 5D. On *Silene* sp., and an unidentified caryophyllaceous species. Iran]	*Aphidura amphorosiphon*
6	Abdominal (or thoracic-abdominal) discal plate present, sometimes divided in transversal bands	7
–	Abdominal discal plate absent; a broken and irregularly edged spinopleural patch usually present, sometimes with bridges to marginal sclerites	8
7	Mesosternal processes wide and low. Longest dorsal setae on abdominal segment 2–4 are 10–11 μm. Discal plate sometimes divided in transversal bands. Siphunculus 1.6–2.0 times cauda, which has 7–11 setae. [Kadyrbekov (2013): fig. 6. On *Melandrium album*. Kazakhstan]	*Aphidura melandrii*
–	Mesosternal processes more or less narrow and tall. Longest dorsal setae on abdominal segment 2–4 are 10–55 μm. Discal plate always complete. Siphunculus 1.6–2.6 times cauda, which has 5–8 setae. [Nieto Nafría et al. (2013): fig. 1A. On *Saponaria* sp., *Silene commutata*, *Silene kuschakewiczii*, *Silene lithophila*, *Silene vulgaris*, *Silene wallichiana*, *Silene wolgensis* and *Silene* sp. Kazakhstan, Pakistan, Tajikistan, and India]	*Aphidura ornatella*
8	Siphunculus 1.7–2.7 times cauda. Longest frontal setae 22–28 μm and 1.0–1.4 times basal diameter of antennal segment III. [Kadyrbekov (2013): fig. 4. On *Gypsophila altissima* and *Gypsophila paniculata*. Kazakhstan]	*Aphidura naimanica*
–	Siphunculus 1.5–1.7 times cauda. Longest frontal setae 35–40 μm and 1.6–1.8 times basal diameter of antennal segment III. [Kadyrbekov (2013): fig. 5. On *Cerastium cerastoides*. Kazakhstan]	*Aphidura alatavica*
9	First segment of tarsi with 4 or less often with 3 setae. [Head and prothoracic transversal band as dark as thoracic-abdominal discal plate. Siphunculi cylindrical and straight. On Rosaceae species]	10
–	First segment of tarsi usually with 3 setae, sometimes with 2; very infrequently with 4	11
10	Antennal segment VI processus terminalis 2.2–2.7 times antennal segment VI base. Ultimate rostral segment with 2–5 accessory setae. Marginal tubercles usually present on abdominal segments 2–4. [Nieto Nafría et al. (2013): fig. 1D. On *Prinsepia sinensis*. Russia: Far Est, Primorsky Krai]	*Aphidura mordvilkoi*
–	Antennal segment VI processus terminalis 2.9–5.2 times antennal segment VI base. Ultimate rostral segment with 8–10 accessory setae. Abdominal marginal tubercles always absent. [Fig. 3, 4. Nieto Nafría et al. (2013): fig. 2A. On *Prunus erythrocarpa*, *Prunus fruticosa*, *Prunus incana*, *Prunus spinosa*, *Prunus tianschanica*, *Prunus triloba*, *Prunus ulmifolia*, *Prunus verrucosa* and *Prunus* sp. Georgia, Kazakhstan, Iran, Uzbekistan, Tajikistan, and Kyrgyzstan]	*Aphidura bozhkoae*
11	Dorsum of thorax and abdomen with setiferous sclerites, similar in size to spiracular sclerites, and sometimes coalescing between them [Fig. 1. On *Cerastium soleirolii*. France: Corsica]	*Aphidura corsicensis* sp. n.
–	Dorsum of thorax and abdomen never with setiferous sclerites; other sclerotization can be present	12
12	Siphunculus slightly swollen with a maximal width close to 1.2 times minimal stem width and 1.6–2.0 times cauda, which is 1.5–1.8 times its basal width and has 7–11 setae; both as dark as head dorsum and thoracic and abdominal sclerotisation (a discal plate can be present). Longest dorsal setae on abdominal segment 2–4 are 10–11 μm and approximately 0.5 times basal diameter of antennal segment III. [Kadyrbekov (2013): fig. 6. On *Melandrium album*. Kazakhstan]	*Aphidura melandrii*
–	Characters not in above combination	13
13	Siphunculus at most 1.95 times cauda (which is short triangular), pale or uniformly dusky and slight swollen. Dorsum of head and mesosternal processes pale. Segmental thoracic and abdominal sclerotisation and pigmentation absent	14
–	Siphunculus at least 1.90 times cauda, both diversely shaped and coloured. Dorsum of head and mesosternal processes pale or pigmented. Thoracic and abdominal segmental sclerotisation and pigmentation rare completely absent	15
14	Siphunculus at least 0.26 mm, 0.6–0.95 times antennal segment III, and 1.7–1.95 times cauda, which is longer than its basal width. Mesosternal processes conspicuous. [Nieto Nafría et al. (2013): fig. 3A. On *Dianthus carthusianorum*, *Dianthus caryophyllus*, *Dianthus commutatus*, *Dianthus monspessulanus*, *Dianthus rupicola*, *Dianthus* sp. and *Silene borysthenica*. Portugal, Spain, France, Switzerland, Italy and Ukraine]	*Aphidura pujoli*
–	Siphunculus shorter than 0.20 mm, 0.41–0.56 times antennal segment III, and 1.7–1.9 times cauda, which is not longer than its basal width. Mesosternal processes sometimes inconspicuous. [Nieto Nafría et al. (2013): fig. 5B. On *Dianthus* sp. Pakistan]	*Aphidura pakistanensis*
15	Antennal segment I at least 1.25 times its maximal width. Longest dorsal setae on abdominal segments 2–4 are 35–55 μm and 1.5–2.0 times basal diameter of antennal segment III. [Discal plate oval and dark; siphunculi weakly ornamented, smooth distad; Nieto Nafría et al. (2013): fig. 2B. On *Silene italica*, *Silene nutans*, perhaps *Silene viscosa*, and *Silene* sp.; France, Italy, Greece]	*Aphidura delmasi*
–	Antennal segment I at most 1.2 times its maximal width. Longest dorsal setae on abdominal segments 2–4 at most 25 μm and 1.2 times basal diameter of antennal segment III	16
16	Abdomen usually with spinopleural patch and separate marginal sclerites; if a discal plate is present then it has irregular margins and frequently there are windows in spinal areas of the thoracic, if integrated, and anterior abdominal segments. Dorsal patch or plate smooth and reticulated. Siphunculi dark brown to black, subcylindrical and usually straight, 1.8–2.0 times cauda, which is broad triangular and has 10–16 setae. Ultimate rostral segment with 6–10 accessory setae. [Nieto Nafría et al. (2013): fig. 2C. On *Silene inaperta*, *Silene italica*, *Silene nutans*, *Silene saxifraga*, *Silene otites*, *Silene vulgaris*, *Silene wolgensis* and *Silene* sp. France, Switzerland, Italy, Hungary, Romania, Ukraine and Russia]	*Aphidura ornata*
–	Characters not in above combination	17
17	Longest setae on abdominal segments 2–4 (dorsum) and antennal segment III 3–8 μm and 0.15–0.50 times basal diameter of antennal segment III	18
–	Longest setae on abdominal segments 2–4 (dorsum) and antennal segment III 8–25 μm and 0.30–1.60 times basal diameter of antennal segment III; if they are 8 μm long then marginal abdominal tubercles present or ultimate rostral segment shorter than second segment of hind tarsi	19
18	Siphunculi dark brown, head dorsum, mesosternal processes and cauda brown to dark brown. Ultimate rostral segment 1.15–1.25 times second segment of hind tarsi. Cauda 1.4–1.8 times its basal width. [Nieto Nafría et al. (2013): fig. 2D. On *Gypsophila paniculata*, *Silene borysthenica*, *Silene moldavica*, *Silene otites*, *Silene wolgensis* and *Silene* sp. Slovakia, Hungary, Greece, Ukraine, and Moldova]	*Aphidura pannonica*
–	Siphunculi (with smoked apex, head dorsum, mesosternal processes and cauda pale. Ultimate rostral segment as long as second segment of hind tarsi. Cauda 1.0–1.1 times its basal width. [Kadyrbekov (2013): fig. 1. On *Gypsophila perfoliata*. Kazakhstan]	*Aphidura togaica*
19	Marginal tubercles on prothorax and abdominal segments 2–4 usually present and spinal tubercle on abdominal segment VIII sometimes present	20
20	Aphids relatively large (longer than 1.7 mm) and provided with an extensive, solid discal plate. Setae on antennal segment III, and on dorsum of head, abdominal segments 2–4 and abdominal segment 8 at least 12, 15, 10 and 27 μm respectively. [Fig. 2. On *Prunus prostrate*. Lebanon]	*Aphidura libanensis* sp. n.
–	Aphids relatively small (shorter than 1.4 mm) with a broken pattern of dorsal sclerotisation. Setae on antennal segment III, and on dorsum of head, abdominal segments 2–4 and abdominal segment 8 at most 8, 10, 8 and 15 μm respectively. [Nieto Nafría et al. (2013): fig. 6A. On *Prunus*. Iran]	*Aphidura iranensis*
–	Marginal and spinal abdominal tubercles absent	21
21	Siphunculi pale, usually as pale as most part of tibiae	22
–	Siphunculi pigmented, usually darker than most part of tibiae	23
22	Antennal segment VI processus terminalis 5.0–5.5 times antennal segment VI base. Cauda triangular or tongue-shaped with slight proximal constriction. Ultimate rostral segment shorter than second segment of hind tarsi. [Nieto Nafría et al. (2013): fig. 6C. On *Gypsophila arenaria*, *Gypsophila paniculata*, *Gypsophila perfoliata*, *Gypsophila* sp. Slovakia, Hungary, Ukraine, Kazakhstan, Russia: Western Siberia]	*Aphidura gypsophilae*
–	Antennal segment VI processus terminalis 2.8–4.0 times antennal segment VI base. Cauda tongue-shaped. Ultimate rostral segment 1.23–1.45 times second segment of hind tarsi. [Clypeus swollen both forward and laterally; Nieto Nafría et al. (2013): fig. 5D. On *Spergularia marina*. Iran]	*Aphidura urmiensis*
23	Cauda tongue-shaped, 1.40–1.80 times its basal width. Mesosternal processes more or less pigmented, usually darker than tibiae. [Thoracic and abdominal sclerotisation variable, usually a spinopleural abdominal patch with irregular edges and windows in several segments, including the posterior ones; siphunculi pigmented, but usually pale than abdominal sclerotized dorsum; Nieto Nafría et al. (2013): fig. 1C. On *Dianthus barbatus*, *Dianthus caryophyllus*, *Dianthus crinitus*, *Dianthus* sp., *Silene conoidea*, *Silene fruticosa*, *Silene italica*, *Silene otites*, *Silene paradoxa*, *Silene thymifolia*, *Silene vulgaris*, and *Silene* sp. Spain, France, Italy, Slovenia, Hungary, Greece, Bulgaria, Turkey, Israel, Iran, Afghanistan, Pakistan, Tajikistan, and Russia: Asiatic]	*Aphidura picta*
–	Cauda triangular, although sometimes with a slight proximal constriction, 1.05–1.40 times its basal width. Siphunculi and mesosternal processes as pale as tibiae	24
24	Ultimate rostral segment 0.90–1.00 times second segment of hind tarsus, with 8–10 accessory setae. Cauda approximately 1.30–1.40 times its basal width. Longest dorsal setae on abdominal segment 2–3 are 8–11 μm and 0.3–0.5 basal diameter of antennal segment III. [Kadyrbekov (2013): fig. 2. On *Silene lithophila*. Kazakhstan]	*Aphidura massagetica*
–	Ultimate rostral segment 1.05–1.45 times second segment of hind tarsus, with 10–16 accessory setae. Cauda approximately 1.05–1.35 times its basal width. Longest dorsal setae on abdominal segment 2–3 are 13–23 μm and 0.6–1.0 basal diameter of antennal segment III. [Nieto Nafría et al. (2013): fig. 4A, B. On *Silene gallica* and *Silene paradoxa*. France]	*Aphidura gallica*

## Supplementary Material

XML Treatment for Aphidura libanensis

XML Treatment for Aphidura corsicensis
